# TKR after posttraumatic and primary knee osteoarthritis: a comparative study

**DOI:** 10.1186/s13018-021-02322-8

**Published:** 2021-03-04

**Authors:** Ong-art Phruetthiphat, Biagio Zampogna, Sebastiano Vasta, Benyapa Tassanawipas, Yubo Gao, John J. Callaghan

**Affiliations:** 1 Department of Orthopedics, Phramongkutklao Hospital and College of Medicine, 315 Ratchvidhi Road, Ratchathewee, Bangkok, 10400 Thailand; 2 Department of Orthopedics and Trauma Surgery, University Campus Bio-Medico of Rome, Rome, Italy; 3 Concord College, Shrewsbury, UK; 4 Department of Orthopedic Surgery and Rehabilitation, University of Iowa Hospital and Clinics, Iowa City, IA USA

**Keywords:** Posttraumatic knee osteoarthritis, Primary knee osteoarthritis, Functional outcome, Mechanical alignment, Total knee replacement, Complication, Aseptic loosening

## Abstract

**Background:**

A few literatures reported that the outcomes of total knee replacement (TKR) in posttraumatic osteoarthritis (PTOA) were lower compared to TKR in primary osteoarthritis (primary OA). The study’s purpose was to compare the comorbidity and outcome of TKR among fracture PTOA, ligamentous PTOA, and primary OA. The secondary aim was to identify the effect of postoperatively lower limb mechanical axis on an 8-year survivorship after TKR between PTOA and primary OA.

**Methods:**

Seven hundred sixteen patients with primary OA, 32 patients with PTOA (knee fracture subgroup), and 104 PTOA (knee ligamentous injury subgroup) were recruited. Demography, comorbidities, Charlson Comorbidity Index (CCI), operative parameters, mechanical axis, functional outcome assessed by WOMAC, and complications were compared among the three groups.

**Results:**

PTOA group was significantly younger (*p*<0.0001) with a higher proportion of men (*p*=0.001) while the primary OA group had higher comorbidities than the PTOA group, including anticoagulant usage (*p*=0.0002), ASA class ≥3 (*p*<0.0001), number of diseases ≥ 4 (*p*<0.0001), and CCI (*p*<0.0001). Both the fracture PTOA group (*p*<0.0001) and ligamentous PTOA group (*p* = 0.009) had a significantly longer operative time than the primary OA group. The fracture PTOA group had significantly lower pain components and stiffness components than the primary OA group. There was no significant difference in the rate of an aligned group, outlier group, and an 8-year survivorship in both groups.

**Conclusion:**

The outcome following TKR in the fracture PTOA was poorer compared to primary knee OA in the midterm follow-up. However, no difference was detected between the ligamentous PTOA and primary knee OA. The mechanical axis alignment within the neutral axis did not affect the 8-year survivorship after TKR in both groups.

**Level of evidence:**

Level III; retrospective cohort study

## Introduction

Fracture around the knee (distal femoral and tibial plateau fracture) can lead to posttraumatic osteoarthritis of the knee (PTOA) [1–4]. Malunion, malalignment, intra-articular osseous defects, retained internal fixation devices, and compromised soft tissues may affect the outcome of total knee replacement (TKR) [1]. On average, patients affected by posttraumatic OA were approximately 10 years younger than those affected by primary knee osteoarthritis of the knee (Primary knee OA) [5, 6]. Additionally, tibial plateau fracture fixation, in older patients, is more likely to require TKR [7]. Only a few literatures reported that surgical challenges and outcomes of TKR in PTOA patients were lower compared to TKR in primary OA [8–12]. However, not much data investigated the patient’s comorbidities, functional outcome, and complication between TKR after PTOA (fracture around the knee and ligamentous injury of the knee) and TKR after primary knee OA.

The purpose of the present study was to compare comorbidity, functional outcome, and complication of TKR among PTOA caused by fracture, PTOA after a ligamentous knee injury, and primary knee OA. The secondary aim was to identify the effect of postoperatively lower limb mechanical axis on the survivorship of total knee replacement between PTOA and primary OA after an 8-year follow-up.

## Material and methods

After the Institutional Review Board Approval, the patient’s medical records were reviewed. Between January 2006 and December 2012, a total of 1225 patients underwent TKRs at our adult reconstruction center. Exclusion criteria were secondary OA knee caused by non-traumatic event (rheumatoid arthritis, osteonecrosis, and septic arthritis), patients with follow-ups less than 2 years, and TKR without patellar resurfacing. Finally, 852 patients undergoing TKRs were recruited in this study. Patients were divided according to OA etiology: 716 patients with primary OA knee, 32 patients with PTOA (knee fracture subgroup), and 104 PTOA (knee ligamentous injury subgroup). Primary OA knee was defined as knee osteoarthritis without a specific cause, while PTOA knee was defined as osteoarthritis of the knee caused by a previous traumatic event. The PTOA group was further divided into 2 subgroups: fracture PTOA subgroup and ligamentous injury PTOA subgroup. Demographic data, comorbidity, ASA (American Society of Anesthesiologists) classification, Charlson Comorbidity Index (CCI) [13], preoperative alignment, preoperative visual analog scale (VAS), preoperative Western Ontario and McMaster Universities Arthritis Index (WOMAC score) [14, 15] were reviewed. Postoperative functional outcomes were assessed with the Western Ontario and McMaster Universities Osteoarthritis Index (WOMAC) [14, 15] with at least a 2-year follow-up. Both of these functional outcome scores were updated prospectively by the patients at their clinical follow-up visits by the completion of kiosk questionnaires. The WOMAC (The Western Ontario and McMaster Universities) is a standardized questionnaire used to evaluate the condition of patients with osteoarthritis of the knee and hip, including 5 items for pain (score range 0–20), 2 for stiffness (score range 0–8), and 17 for functional limitation (score range 0–68). Each component is directly converted into a 0–100 scale, assuming that each question carries equal weight. A score of zero is equivalent to a maximum disability, and a score of 100 is equivalent to no disability. The median follow-up period was 8.0 years (2.0-11.4 years) in the primary OA group and 8.1 years (5.3-11.3 years) in the PTOA group.

### Surgical procedure

Before TKR, all patients received a standardized preoperative radiographic evaluation, including anteroposterior and lateral radiographs, a merchant view, and a full-length standing hip-to-ankle radiograph. A standard medial parapatellar approach was routinely performed. In the standard group, both femoral and tibial components were cemented, and the patella was resurfaced. Depending on the ligament balance after bone cuts, a condylar TKR with a posterior cruciate ligament–substituting insert was used. If necessary, in the PTOA group, intercondylar stabilizing or rotating-hinge TKR were used, and patellar resurfacing was performed. Operation time was defined as the time from incision to suture, and operative blood loss was recorded.

### Postoperatively mechanical alignment

Hip-knee-ankle radiographs (long film study) were available in some patients for postoperatively mechanical axis assessment: 129 patients with primary OA and 122 patients with PTOA. The postoperative knee alignment was classified into two groups; an aligned group defined as postoperative alignment within a neutral mechanical axis (0±3°) [16] while an outlier group defined as the mechanical axis deviated from neutral by >3°. The 8 years follow-up free from aseptic loosening were compared between groups.

### Data management and statistical analysis

Data description was based on means and standard deviation for continuous variables and absolute and relative frequencies for categorical variables. A standard Student’s *t* test was used for continuous variables, while the chi-squared was applied for categorical variables. ANOVA was applied for comparison among primary OA knee, fracture PTOA subgroup, and ligamentous injury PTOA subgroup. Statistical analysis was performed using SAS 9.3 (SAS Institute, Cary, NC) with statistical significance set to *p* < 0.05.

## Result

Our study reported a significant difference in age, gender, and obesity between posttraumatic OA knee (PTOA) and primary OA knee. PTOA patients were significantly younger (56.5 vs. 63.8 years; *p*<0.0001) at the time of surgery, with a higher proportion of men (51.5% vs. 36.9%; *p*=0.001) compared with the primary OA group. On the contrary, the primary OA group undergoing TKR predominantly in females, with a significantly higher obesity rate than the PTOA group. However, smoking and alcoholic drinking had no significant difference in both groups (Table 1). Additionally, the Primary OA group had higher comorbidities than PTOA group including anticoagulant usage (51% vs 30.9%, *p*=0.0002), ASA class ≥3 (38.8% vs 21.6%, *p*<0.0001), number of disease ≥ 4 (69.6% vs 45.3%, *p*<0.0001), and Charlson Comorbidity Index (3.6 vs 2.8, *p*<0.0001) (Table 1).

**Table 1:** Patient’s demographic data and comorbidity between posttraumatic osteoarthritis of the knee (PTOA) and primary osteoarthritis of the knee (primary OA)

Demographic data	PTOA	Primary OA	*p* value
Age^a^ (years)	56.5 (31.0-82.0)	63.8 (30.0-95.0)	**<0.0001**
Gender^b^			
Male	70 (51.5%)	264 (36.9%)	**0.001**
Female	66 (48.5%)	452 (63.1%)	
BMI^c^			
Normal (18 to <25 kg/m^2^)	14 (10.4%)	42 (5.9%)	**0.025**
Overweight (25 to <30 kg/m^2^)	37 (27.4%)	128 (17.9%)	
Obesity (>30 kg/m^2^)	84 (62.7%)	544 (**76.0%**)	
Smoking^b^	8 (5.9%)	35 (4.9%)	0.627
Alcohol drinking^b^	59 (43.4%)	255 (35.6%)	0.085
Comorbidity	PTOA	Primary OA	*p* value
Anticoagulant^b^			
Yes	42 (30.9%)	365 (51.0%)	**0.0002**
No	94 (69.1%)	351 (49.0%)	
ASA classification^c^			
1	7 (5.2%)	11 (1.6%)	**<0.0001**
2	98 (73.1%)	424 (59.6%)	
≥3	29 (21.6%)	276 (38.8%)	
Number of diseases^b^			
1-3	70 (54.7%)	214 (30.4%)	**<0.0001**
≥4	58 (45.3%)	488 (69.6%)	
Charlson Comorbidity Index^a^	2.8 ± 1.4 (0.0-7.0)	3.6 ± 1.5 (0.0-10.0)	**<0.0001**

^a^Mean, range

^b^*n* percentage

^c^The values are given as the number of patients, with the percentage in parentheses

Statistical significance at *p*<0.05

### Perioperative outcomes

Both fracture PTOA group (125.3 vs 100.1, *p*<0.0001) and ligamentous PTOA group (106.5 vs 100.1, *p* = 0.009) had significantly longer operative time than the primary OA group, while there were no differences in operative blood loss (175.9 vs 118.5, *p* = 0.06; 123.5 vs 118.5, *p* = 0.94) and LOS among three groups (3.1 vs 2.9, *p* = 0.63; 2.6 vs 2.9, *p* = 0.12) (Table 2). In addition, the preoperative visual analog scale (VAS) was comparable among the three groups. Postoperative VAS in fracture PTOA was significantly higher than the primary OA group, while there was no significant difference in pain score between the ligamentous PTOA subgroup and the primary OA group (Table 3).

**Table 2:** Operative data and length of hospital stay among posttraumatic OA knee (fracture PTOA and ligamentous PTOA) and primary OA knee

Operative data	Mean ± SD (range)	*n* (%)	*p* value
**Require femoral osteotomy before TKA**			N/A
**Fracture PTOA (*n*=32)**		3	
**Ligamentous PTOA (*n*=104)**		0	
Primary OA (*n*=716)*		0	
**Operative time (minutes)**			
Fracture PTOA (*n*=32)	125.3±48.0 (75-290)		**<0.0001**
Ligamentous PTOA (*n*=104)	106.5±22.3 (50-202)		**0.009**
Primary OA (*n*=716)*	100.1±18.8 (48-197)		
**Blood loss (milliliters)**			
Fracture PTOA	175.9±130.7 (50-500)		0.06
Ligamentous PTOA	123.5±96.3 (25-500)		0.94
Primary OA*	118.5±139.5 (25-270)		
**Length of stay (days)**			
Fracture PTOA	3.1±2.1 (1.0-13.0)		0.63
Ligamentous PTOA	2.6±1.2 (2.0-12.0)		0.12
Primary OA*	2.9±1.2 (1.0-12.0)		

*Reference; significant at *p*<0.05

**Table 3:** Preoperative and postoperative WOMAC and VAS among posttraumatic OA knee (fracture PTOA and ligamentous PTOA) and primary OA knee

**Preoperative WOMAC**	**Mean ± SD**	**Range**	***p*** **value**
**Pain component**			
**Fracture PTOA**	40.5 ± 30.6	0.0-100.0	0.20
**Ligamentous PTOA**	43.6 ± 19.7	0.0-100.0	0.11
Primary OA*	48.7 ± 21.9	0.0-100.0	
**Stiffness component**			
**Fracture PTOA**	36.4 ± 30.6	0.0-100.0	0.70
**Ligamentous PTOA**	39.4 ± 21.3	0.0-87.5	0.95
Primary OA*	40.2 ± 21.6	0.0-100.0	
**Functional component**			
**Fracture PTOA**	41.2 ± 24.2	5.7-87.5	0.39
**Ligamentous PTOA**	45.3 ± 21.4	0.0-95.5	0.80
Primary OA*	46.7 ± 19.0	0.0-97.7	
**Postoperative WOMAC**	**Mean ± SD**	**Range**	***p*** **value**
**Pain component**			
Fracture PTOA	70.0±25.0	25.0-100.0	**0.007**
Ligamentous PTOA	80.2±23.8	0.0-100.0	0.16
Primary OA*	85.7±19.6	10.0-100.0	
**Stiffness component**			
Fracture PTOA	50.0±25.0	0.0-100.0	**0.013**
Ligamentous PTOA	63.2±22.8	0.0-100.0	0.98
Primary OA*	63.8±18.3	0.0-100.0	
**Functional component**			
Fracture PTOA	60.9±21.0	22.7-89.8	0.08
Ligamentous PTOA	73.6±20.6	18.2-97.7	0.83
Primary OA*	71.9±19.7	3.4-100.0	
**Visual analog pain score**	Mean ± SD	Range	*p* value
**Preoperative VAS**			
Fracture PTOA	8.3 ± 1.4	6.0-10.0	0.64
Ligamentous PTOA	8.0 ± 1.3	4.0-10.0	0.99
Primary OA*	8.0 ± 1.3	3.0-10.0	
**Postoperative VAS**			
Fracture PTOA	2.1 ± 1.8	0.0-6.0	**0.019**
Ligamentous PTOA	1.7 ± 1.8	0.0-8.0	0.063
Primary OA*	1.3 ± 1.7	0.0-10.0	

*Reference; significant at *p*<0.05

### Clinical outcomes

There were no differences in all three components of the preoperative WOMAC score among posttraumatic OA knee (fracture PTOA and ligamentous PTOA) and primary OA knee (Table 3). Besides, the fracture PTOA group had significantly lower pain components and stiffness components than the primary OA group for the postoperative outcome. However, there were no differences in all components of the WOMAC score between ligamentous PTOA and the primary OA group (Table 3).

### Radiographic outcomes and 8-year survivorship

Postoperatively radiographic assessment defined that they were not significantly different in mechanical axis (0.4 vs. 0.9, *p* = 0.263) between groups, except PTOA group had a more posterior slope than the primary OA group (2.9 vs. 2.1, *p* = 0.018) (Table 4). There was no significant difference in the rate of an aligned group (33.3% vs. 29.5%, *p*=0.514), including the outlier group (66.7% vs. 70.5%) between primary OA and PTOA groups. Moreover, there was no difference in 8-year survivorships free from aseptic loosening in both groups. There was no aseptic loosening in primary OA, and there was only one aseptic loosening from the PTOA group at 8-year follow-up.

**Table 4:** Radiographic parameters between posttraumatic OA and primary OA knee

Radiographic alignment	PTOA	Primary OA	*p* value
**Mechanical axis**			
Preoperative^a^	3.2 ± 10.4 (−28.0 to 34.0)	4.0 ± 9.4 (−27.0 to 34.0)	0.570
Postoperative^a^	0.4 ± 3.7 (−8.0 to 10.0)	0.9 ± 3.6 (−12.0 to 18.0)	0.263
Postoperatively mechanical axis			0.514
An aligned group^b^	36 (29.5%)	43 (33.3%)	
An outlier group^b^	86 (66.7%)	86 (70.5%)	
**Posterior slope**^a^			
Preoperative	8.1 ± 3.5 (0.0 to 19.0)	7.2 ± 4.1 (−3.0 to 23.0)	0.052
Postoperative	2.9 ± 3.2 (−6.0 to 11.0)	2.1 ± 2.2 (−2.0 to 12.0)	**0.018**

^a^Mean, range; In AP X-ray (+ = varus knee, − = valgus knee)

^b^Number (percentage), In AP X-ray. An aligned group defined as a neutral mechanical axis (0±3°) while an outlier group defined as the mechanical axis deviated from neutral by >3°

In lateral X-ray (+ = posterior slope, − = anterior slope)

### Complications

There was no difference in postoperative complications (surgical site infection, urinary tract infection, venous thrombotic event), including readmission within 90 days (Table 5).

**Table 5:** Complications including readmission between posttraumatic (PTOA) and primary OA knee

Parameters	PTOA (***n***=136)	Primary OA (***n***=716)	***p*** value
Complications^b^			0.262
Surgical site infection^a^	2 (1.5%)	2 (0.3%)	
Urinary tract infection^a^	0 (0%)	1 (0.1%)	
VTE event	1 (0.7%)	1 (0.1%)	
No complication^a^	133 (97.8%)	712 (99.5%)	
Readmissions^a^			
Within 30 days	2 (1.5%)	21 (2.9%)	0.561
Between 30 and 90 days	2 (1.5%)	15 (2.1%)	1.000
Need for revision TKA	1 (0.7%)	0 (0%)	

^a^*n* percentage

^b^The values are given as the number of patients, with the percentage in parentheses

Statistical significance at *p*<0.05

## Discussion

Previous studies demonstrated that TKR after PTOA had lower functional outcomes than TKR in primary OA [8–12]. However, not much data described the patient’s comorbidities, functional outcome, and complication between PTOA (fracture around the knee and ligamentous injury of the knee) and primary OA of the knee. Our study firstly defined the comparison of functional knee outcomes among three groups (fracture PTOA, ligamentous PTOA, and primary OA). Secondly, this study also demonstrated a comparison of radiologic outcomes, including their 8-year survivorship between posttraumatic OA and primary OA following TKR.

J. Dexel et al. demonstrated that patients with PTOA were significantly younger at the time of surgery than those with primary OA (62 vs. 71 years, *p*<0.001). In addition, operative time was significantly longer for both of the PTOA group compared with primary OA (*p*<0.001) [6]. This study was similar to the previous study [6] to both ages when performed TKA (56.5 vs. 63.8 years, *p*<0.0001), including the operative time (125.3 vs. 100.1 min, *p*<0.0001 and 106.5 vs. 100.1 min, *p*=0.009).

K.J. Saleh et al. [17] showed the improvement of knee score from 51 to 80 degrees and knee range of motion (ROM) from 87 to 105 degrees, preoperative and postoperative, respectively in TKA with the previous history of open reduction and internal fixation of fractures of the tibial plateau. E.C. Papadopoulos et al. [1] highlighted the improvement of Knee Society Scores from 48 to 66 degrees and knee ROM from 83 to 89 degrees, preoperative and postoperative, respectively in TKR following a prior distal femoral fracture. In addition, J-W Wang et al. demonstrated the improvement of knee score from 28 to 87 degrees and knee range of motion from 78 to 104 degrees, preoperative and postoperative, respectively and they concluded that TKR with intraarticular bone resection is useful for extra-articular deformity of femur less than 20 degrees and tibia deformity less than 30 degrees [18].

A previously prospective study defined that there were no significant differences in knee score (88 vs. 92), WOMAC (75 vs. 79), SF12 (41 vs. 45; 40 vs. 44), or range of motion (95 vs. 99 degrees) between PTOA (*n*=29) and primary OA (*n*=58) after TKR, except the PTOA group had significantly higher complication rate than primary OA group (13.7% vs. 0%, *p*=0.01) [19]. This study firstly defined the comparison of functional knee outcomes among three groups (fracture PTOA, ligamentous PTOA, and primary OA). The fracture PTOA group had significantly lower pain components and stiffness components than the primary OA group. Simultaneously, there were no differences in all components of the WOMAC score between ligamentous PTOA and the primary OA group.

B.S. Brocker et al. identified a comparison study between PTOA and primary OA undergoing TKA using the NIS database (National Inpatient Sample), and they demonstrated that patients with primary OA had a higher prevalence of obesity, diabetes, heart disease, and lung disease [20]. This study was comparable with the NIS database in that the primary OA group had higher comorbidities, including higher CCI (*p*<0.0001).

This study showed no difference in surgical site infection in both groups (1.5% vs. 0.3%, *p*=0.262) even though there was the statistical significance of operative time among the three groups. Previous studies defined that PTOA had a significantly higher rate of superficial wound infection than primary OA following TKR (*p*<0.001) [20] and the PTOA group had a significantly higher likelihood of wound complication than those with primary OA patients (odd ratio 1.8 with 95% confidential interval between 1.55 and 2.09, *p*<0.001) [21]. In addition, Peersman et al. demonstrated a longer operative time related to surgical site infection in TKR [22]. The result from this study was different from previous literature because the PTOA group mainly came from the ligamentous PTOA (*n*=104; 76.5%), which only had a small surgical scar from a previous ligamentous knee reconstruction without any difference in surgical site infection between groups.

Previous studies demonstrated that failure to restore a neutral mechanical axis is related to increased component loosening and lower long-term prosthesis survival [23–27]. Computer-navigated TKR provides more accuracy of component position [28–30]. However, an ideal target for alignment remains an issue for debate. Another data defined that postoperative mechanical axis within a neutral mechanical axis (0±3°) did not improve the 15-year implant survival rate following modern TKR [16]. They showed a weak relationship between the survival of primary TKR and mechanical axis alignment at 15-year follow-up [31]. However, current studies have not identified the association of mechanical axis and aseptic loosening between PTOA and primary OA. This study has first defined the mechanical axis’s correlation assessed by long leg alignment radiographs and 8-year survivorship from aseptic loosening in both groups. Our study showed no relationship between the postoperative alignment within a neutral axis and 8-year survivorship in both PTOA and primary OA underwent TKR.

### Study limitation

Our study presented several limitations starting from the retrospective nature. Second, identification of complications depends on the existing medical records; for example, comorbidities documented are influenced by the treating physician, so information regarding patient systemic illness may be incomplete. This would affect our calculation of the CCI score for patients. Third, our sample size was small compared with large national studies, so we may have failed to detect subtle statistically significant differences in the multiple parameters analyzed. Fourth, even though this study firstly demonstrated the medium to long-term follow-up of the association of mechanical axis and aseptic loosening between PTOA and primary OA, the PTOA group database mainly came from the ligamentous injury subgroup. Further studies are needed for the identification of this correlation, focusing on fracture PTOA. Last is the retrospective data analysis.

## Conclusion

The outcome following TKR in the fracture PTOA group was poorer than the knee’s primary osteoarthritis in the midterm follow-up. However, no difference was detected between the ligamentous PTOA and primary knee OA. The mechanical axis alignment within the neutral axis did not affect the 8-year survivorship after TKR in both primary OA and PTOA.

## Data Availability

The datasets used and/or analyzed during the current study are available from the corresponding author on reasonable request.

## Abbreviations

ASA: American Society of Anesthesiologists classification
CCI: Charlson Comorbidity Index
VAS: Visual analog scale
WOMAC: Western Ontario and McMaster Universities Arthritis Index
PTOA: Posttraumatic osteoarthritis
Primary OA: Primary osteoarthritis
TKR: Total knee replacement
